# Complete resolution of urinary incontinence with treatment improved the health-related quality of life of children with functional daytime urinary incontinence: a prospective study

**DOI:** 10.1186/s12955-020-1270-2

**Published:** 2020-01-21

**Authors:** Hirokazu Ikeda, Chisato Oyake, Yuta Oonuki, Masaki Fuyama, Tsuneki Watanabe, Takashi Kyoda, Setuko Tamura

**Affiliations:** 1 Department of Pediatrics, Showa University Fujigaoka Hospital, Yokohama, Japan; 2 Faculty of Psychology, Tokyo Seitoku University, Tokyo, Japan

**Keywords:** Quality of life, Daytime urinary incontinence, Improvement

## Abstract

**Background:**

To assess the health-related quality of life (HRQOL) of children with daytime urinary incontinence (DUI) based on pre- and post-treatment self-reports and parent proxy-reports.

**Methods:**

The study population comprised 117 children with at least one episode of DUI per week and their caregivers as well as 999 healthy children (control group). The Pediatric Quality of Life Inventory 4.0 (PedsQL) questionnaire was administered to assess the HRQOL of children. To assess the degree of improvement in HRQOL, we categorized children into two groups: group A achieved complete response (CR) to treatment within 12 months and group B did not achieve CR within 12 months. CR was defined as the complete resolution of symptoms or alleviation of symptoms to < 1 DUI episode/month.

**Results:**

Valid responses were collected from 84 children [53 boys and 31 girls; mean age: 7.9 ± 1.5 years (range, 6–12)]. Sixty-two patients (73.8%) were classified into group A and 22 (26.1%) into group B. Based on self-reports, significant post-treatment improvement was observed in the scores of all PedsQL items (mean total score: 82.2 ± 11.3 vs. 87.2 ± 9.8; *P* = 0.003). Group A showed significant improvement in the scores of all PedsQL items after achievement of CR based on child self-reports; however, this was improvement not observed in group B.

**Conclusions:**

To the best of our knowledge, this is the first study to demonstrate the complete resolution of DUI with treatment for improving the HRQOL of these children.

## Background

Daytime urinary incontinence (DUI) is defined as involuntary leakage of urine among children aged > 5 years during daytime activities such as in school classrooms or on playgrounds [1]. The reported prevalence of at least one DUI episode per week among children aged 7, 11–13, and 15–17 is 2.5, 0.5, and 0.3%, respectively [2–4]. The reported rate of reduction in at least one DUI episode per week in children aged 7–17 years is 0.2% per year [5]. As DUI occurs in everyday life, children with this condition are under continuous stress until the alleviation of symptoms. Therefore, DUI treatment should aim not only to cure the symptoms but also to eliminate suffering related to the condition as only a limited proportion of patients achieve spontaneous remission.

According to the International Children’s Continence Society (ICCS), standard urotherapy should be offered as the first-line treatment for all types of DUI in children aged > 5 years [6]. Behavioral modifications such as timed voiding, the avoidance of maintaining maneuvers, and optimal voiding posture are essential aspects of standard urotherapy [1]. This urotherapy additionally includes lifestyle advices for parents and caregivers as it is necessary for their to understand treatment methods and as their cooperation is indispensable to achieve behavioral modification in children [7]. Thus, it is important to know how accurately parents and caregivers assess the quality of life and the psychosocial problems of children with DUI.

Although DUI has a negative impact on the psychological health of the affected children, there is no consensus on the consequences of DUI on the psychosocial functioning of children after remission of the disease [8, 9]. Assessment of health-related quality of life (HRQOL) is essential to understand the impact of chronic diseases; in addition, it is important to assess the post-treatment improvement in patient functioning as well as the sense of well-being of children and their families [10–12]. Friends and school life have a key impact on the HRQOL of school-going age children. Children cannot easily conceal DUI in their daily life; thus, DUI is negatively associated with social and school functioning and the overall HRQOL [13–16]. In addition, DUI tends to affect every aspect of the affected child’s life, including the future occupation and social relationships [17, 18]. The impact of psychosocial problems, including social and school functioning, on children with DUI has not been studied as extensively as in nocturnal enuresis [19, 20]. In addition, little is known about the HRQOL of children with DUI not only after treatment [21–23], but after achievement of complete response (CR) to treatment. Owing to the very nature of the condition, children affected by DUI are subject to ridicule and teasing by their friends and teachers [24]. Therefore, even if the frequency of episodes of DUI decreases, lack of complete disappearance of DUI is believed to have a continuing impact on the child’s HRQOL. In the present study, we assessed the HRQOL of children with DUI based on both child self-reports and parent proxy-reports, before and after achievement of CR to treatment. The study hypotheses were: 1) HRQOL is more impaired in children with DUI than healthy children. 2) The child self-reported and parent proxy-reported HRQOL of children with DUI will improve after achievement of CR compared to the pre-treatment level. 3) The child self-reported and parent proxy-reported HRQOL of children with DUI who achieve CR is not different from that of healthy children.

## Methods

### Study Population

A total of 173 children in the age-group of 5–16 years who presented at our outpatient clinic between April 2012 and March 2015 and who had at least one episode of DUI per week were enrolled in this study along with their caregivers. These children were followed up for at least 12 weeks. The inclusion criterion was diagnosis of functional DUI with or without nocturnal enuresis according to the ICCS criteria (minimum age: 5 years) [1]. The exclusion criteria included, diagnosis of neurogenic bladder, behavioral disorders (such as attention deficit hyperactivity disorder, autism spectrum disorder), developmental delay (intelligence quotient < 70), concomitant chronic disease that can affect the quality of life, or presence of another chronic medical condition requiring daily medication. Children with congenital urethral anomalies were also excluded.

At initial presentation, all children underwent physical examination, sonography, and uroflowmetry. Parents completed a voiding questionnaire and a 48-h bladder diary for their children. The frequency of DUI episodes before and during treatment as well as the treatment effects were assessed according to the ICCS criteria. CR was defined as complete remission of symptoms or less than one symptom episode per month [1] (the definition of complete response was updated to 100% reduction of symptom based on pre-treatment baseline of the frequency of symptoms according to the standardization of terminology of lower urinary tract function in children and adolescents in 2017 [6]). After the initial visit, standard urotherapy according to the ICCS guideline [1], including timed voiding, optimal voiding posture, and constipation therapy, was initiated in all cases; in the absence of any therapeutic response after 4 weeks, antimuscarinic therapy (with solifenacin or propiverine) was administered. The control group consisted of 999 typically developing children in the same age-group. The control group was mainly recruited from two elementary schools: one in the south part of Tokyo and the other in the north part of Tokyo. The 84 patients were classified into two groups based on the time required to achieve CR. Group A included children who achieved CR within 12 months after initiation of first-line therapy, while group B included children who did not achieve CR within 12 months.

The present study was approved by the institutional review board of the Showa University Fujigaoka Hospital (2012122) and the Tokyo Seitoku University (15–4).

### Assessment instrument

The Pediatric Quality of Life Inventory 4.0 (PedsQL) Generic Core Scales [25], the Japanese version [26] was administered to patients, their caregivers, and healthy controls at the two local elementary schools. PedsQL consists of a child self-report form and a parallel parent proxy-report form for age groups 5–7 years, 8–12 years, and 13–18 years. The parent proxy-report assesses the caregiver’s perceptions of their child’s HRQOL. Children completed the child self-reports independently; impartial assistance was provided by the attending physician to any child who had difficulty in comprehending the questions. The caregivers completed the parallel parent proxy-reports consisting of nearly identical items. The PedsQL is a 23-item questionnaire encompassing physical health (eight items), emotional functioning (five items), social functioning (five items), and school functioning (five items). Items are rated on a five-point scale ranging from 0 (“never a problem”) to 4 (“almost always a problem”). Items are reverse scored and transformed linearly from 0 to 100 (0 = 100, 1 = 75, 2 = 50, 3 = 25 and 4 = 0), with higher scores indicating better HRQOL. The physical health summary score is the same as the physical functioning scale score. The psychosocial health summary score is calculated as the mean score of the items in the emotional, social, and school functioning scales.

### Outcome measures

**Fig. 1:**
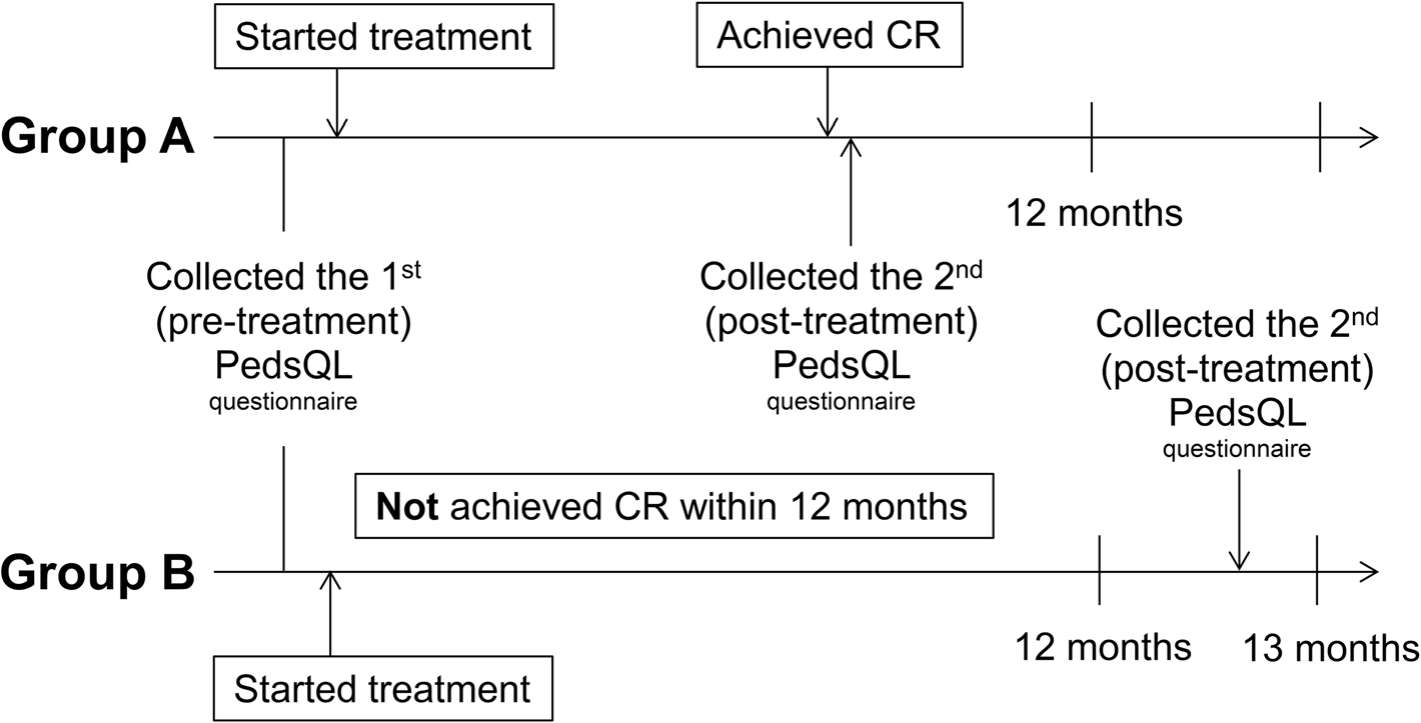
Time-Points for data collection using the PedsQL questionnaire

A schematic illustration of the timing of data collection is presented in Fig. 1. Prior to initiation of treatment, both the child self-reported and the parent proxy-reprted HRQOL were assessed by means of the PedsQL (1st PedsQL). If children with DUI achieved CR within 12 months of treatment initiation (group A), they recompleted the PedsQL at the time of achievement of CR (2nd PedsQL). If children with DUI did not achieve CR within 12 months of treatment initiation (group B), they recompleted the PedsQL 12 to 13 months after the start of treatment (2nd PedsQL).

### Data analysis

Data analysis was performed using JMP Pro 13 (SAS Institute JaPan, Co., Ltd., Tokyo, Japan). The unpaired *t*-test was used to assess differences between patients and healthy controls with respect to physical health summary, psychosocial health summary, and total PedsQL scores. Pre-treatment scores were compared with scores obtained after achievement of CR using independent sample *t*-test. The magnitude of any differences was calculated as an effect size {(mean post-treatment − mean pre-treatment) / pooled SD] with 95% confidence intervals. Effect sizes are usually categorized as follows: small (0.20–0.49), medium (0.50–0.79), and large effects (≥0.80) [27]}. Data are exPressed as mean ± standard deviation (SD). All *P* values less than 0.05 were considered indicative of statistical significance.

## Results

### Study participants

**Fig. 2:**
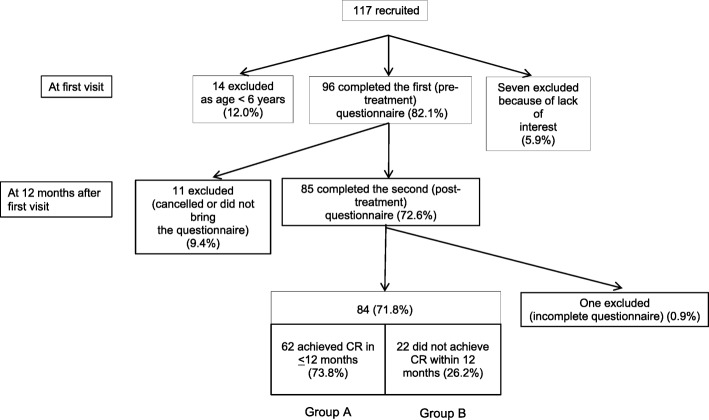
Schematic illustration of the study design and patient-selection criteria

A schematic illustration of the study design and patient-selection criteria is presented in Fig. 2. A total of 117 consecutive children aged 5–12 years who Presented at our institution with at least one episode of DUI from April 2012 to March 2015 were recruited for this study. Seven families (5.9%) declined to participate because of a lack of interest in the study. Fourteen (12.0%) families were excluded because the child was aged < 6 years and the PedsQL is designed for subjects of school-going age. Of the remaining 96 patients, 85 returned valid PedsQL questionnaire responses after achieving CR within 12 months or 12–13 months after treatment initiation; 84 of these 85 patients completed the PedsQL both before treatment (1st PedsQL) and after CR within 12 months or 12–13 months after initiation of treatment (2nd PedsQL) and were included in the final analyses. Of the 84 patients who achieved CR, 18 received standard urotherapy as first-line therapy, 17 received a combination of standard urotherapy and propiverine, and 49 received a combination of standard urotherapy and solifenacin as second-line therapy.

### Characteristics of the study population

The demographic and clinical characteristics of subjects are summarized in Table 1. The mean age of 84 patients (53 boys, 31 girls) was 7.9 ± 1.5 years (range, 6–12). Nearly all caregivers who completed the PedsQL questionnaire were mothers (96.4%). In the healthy control group, 450 of 999 (45.0%) were male and the median years of elementary school was third grade. There were no significant differences between the patient group and the healthy control group in terms of sex (*P* = 0.125). The median grade in elementary school was significantly lower in the patient group than in the healthy control group (*P* < 0.001). Sixty-two patients (73.8%) were classified into group A (achieved CR within 12 months) and 22 Patients (26.1%) were classified into group B (did not achieve CR within 12 months). The mean time to achieve CR in group A was 6.4 ± 2.2 months (median: 6 months).

**Table 1:** Characteristics of children with daytime urinary incontinence

Variable	*N* = 84
Age (years)	
Mean (SD)	7.9 (1.5)
Range	6–12
Sex	
Female	31
Male	53
Percentage of Patients who achieved CR	
Within 12 months or less (grouP A)	62 (73.8%)
not achieved CR during study Period (grouP B)	22 (26.1%)
Duration of CR in grouP A (months)	
Mean (SD)	6.4 (2.2)
Range	3–12
DVSS	
Mean (SD)	10.4 (2.6)

### Comparison of patients and healthy controls (Table 2)

#### Child self-reported HRQOL

The mean PedsQL total scale score based on the child self-reports was not significantly different between the patient group and the healthy control group before treatment (82.2 ± 11.3 vs. 83.6 ± 12.6; *p* = 0.108). In the patient group, the mean PedsQL total scale score based on the child self-reports significantly improved after treatment compared with the pre-treatment score (82.2 ± 11.3 to 87.2 ± 9.8; *p* = 0.003). Although there were no significant differences between the patient group and the healthy control group with respect to pre-treatment physical health summary scores, physical health summary score in the patient group significantly improved after treatment (88.9 ± 10.2 vs. 92.9 ± 8.7; *p* = 0.006). The mean pre-treatment psychosocial health summary score in the patient group was significantly lower than that in the healthy control group (80.0 ± 12.9 vs. 83.4 ± 13.2; *p* = 0.015). In the patient group, the psychosocial health summary score significantly improved from 80.0 ± 12.9 before treatment to 85.3 ± 11.4 after treatment (*p* = 0008). This indicated impaired psychosocial quality of life of patients with DUI and a greatly improved psychosocial quality of life among patients who received treatment for DUI. The patient group had a significantly lower social functioning domain score (82.9 ± 15.4 vs. 88.9 ± 15.7; *p*< 0.001) as compared to the healthy control group. There was a significant improvement in the emotional functioning domain score (73.7 ± 17.3 to 79.6 ± 17.4; *p* = 0.022), the social functioning domain score (82.9 ± 15.4 to 87.9 ± 13.8; *p* = 0.032), and the school functioning domain score (83.5 ± 14.5 to 88.5 ± 11.2, *p* = 0.025) in the patient group after treatment compared with the respective pre-treatment scores.

**Table 2:** Comparison of mean PedsQL scores between cases and controls in different functioning categories

	Child Self-Report					Parent-Proxy report				
	Healthy controls (*N* = 999)	Cases (*N* = 84)				Healthy controls (*N* = 999)	Cases (*N* = 84)			
		Pre-treatment	*p* value: Cases vs. Healthy controls	Post-treatment	*p* value: Pre-treatment vs. Post-treatment		Pre-treatment	*p* value: Cases vs. Healthy controls	Post-treatment	*p* value: Pre-treatment vs. Post-treatment
Total score	83.6 ± 12.6	82.2 ± 11.3	0.108	87.2 ± 9.8	0.003	85.5 ± 13.4	85.4 ± 11.9	0.583	89.8 ± 10.3	0.014
Physical Health Domain	85.2 ± 14.8	88.9 ± 10.2	0.118	92.9 ± 8.7	0.006	86.2 ± 19.9	89.2 ± 14.3	0.436	94.6 ± 8.6	0.047
Psychosocial Health Domain	83.4 ± 13.2	80.0 ± 12.9	0.015	85.3 ± 11.4	0.008	85.2 ± 13.2	84.1 ± 13.0	0.387	88.2 ± 11.63	0.033
Emotional Functioning	76.5 ± 18.9	73.7 ± 17.3	0.129	79.6 ± 17.4	0.022	84.5 ± 14.9	82.4 ± 15.1	0.205	88.6 ± 11.12	< 0.001
Social Functioning	88.9 ± 15.7	82.9 ± 15.4	< 0.001	87.9 ± 13.8	0.032	86.5 ± 17.4	84.3 ± 20.4	0.517	87.6 ± 16.6	0.324
School Functioning	84.9 ± 13.6	83.5 ± 14.5	0.445	88.5 ± 11.2	0.025	84.6 ± 14.2	85.6 ± 13.5	0.596	88.3 ± 12.9	0.161

#### Parent proxy-reported HRQOL

No significant difference was observed between the patient group and the healthy control group with respect to pre-treatment mean PedsQL total scale score based on parent proxy-reorts (85.4 ± 11.9 vs. 85.5 ± 13.4; *P* = 0.583). Similarly, no significant difference was observed between the patient group and the healthy control group with respect to the mean physical health summary score and the mean psychosocial health summary score. Therefore, based on the parent proxy-reports, there was no significant difference between the patient group and the healthy control group with respect to pre-treatment emotional functioning domain, social functioning domain, or school functioning domain scores. However, significant post-treatment improvement was observed in the mean PedsQL total scale score (85.4 ± 11.9 to 89.8 ± 10.3; *P* = 0.014), physical health summary score (89.2 ± 14.3 to 94.6 ± 8.6; *P* = 0.047), and psychosocial health summary score (84.1 ± 13.0 to 88.2 ± 11.6; *P* = 0.033). With respect to domain scores, no significant improvement was observed in the social functioning and school functioning domain scores; however, there was a significant improvement in the emotional functioning domain score after treatment compared with pre-treatment score (82.4 ± 15.1 to 88.6 ± 11.1; *P* < 0.001).

#### Improvement in HRQOL in groups A and B

In group A, the mean PedsQL total scale score (81.9 ± 11.6 vs. 88.5 ± 10.1; *p* = 0.001, effect size 0.6), physical health summary score (88.4 ± 10.8 vs. 93.8 ± 8.4; *p* = 0.003, effect size 0.56), psychosocial health summary score (79.7 ± 13.2 vs. 86.7 ± 11.7; *p* = 0.003, effect size 0.56) as well as scores for emotional, social, and school functioning based on child self-reports were significantly improved after achievement of CR compared to the respective pre-treatment scores (Table 3). This indicated impaired HRQOL of patients with DUI and considerable improvement in HRQOL after achievement of CR. Although the pre-treatment mean psychosocial health summary score and social functioning domain score in group A were significantly lower than those in the healthy control group, the scores were significantly higher after achievement of CR. Furthermore, in group A, the mean PedsQL total scale score and the physical health summary score after achievement of CR were significantly higher than those in the healthy control group. There was no significant difference between patient groups A and B with respect to pre-treatment PedsQL total scale score, psychosocial health summary score, emotional functioning domain score, or social functioning domain based on parent proxy-reports (data not shown). In addition, no significant improvement was observed in group B with respect to mean PedsQL total scale score, physical health summary score, psychosocial health summary score, or the other functioning domain scores based on child self-reports.

**Table 3:** Pre- and Post-treatment mean PedsQL scores of patients in groups A and B and of healthy controls: child self-report

	Healthy controls (N = 999)	Group A (*N* = 62)					Group B (*N* = 22)				
		Pre-treatment	Pre-treatment vs. Healthy controls	After CR	After CR vs. Healthy controls	After CR vs. Pre-treatment	Pre-treatment	Pre-treatment vs. Healthy controls	12 months after initiation of treatment	12 months after initiation of treatment vs. Healthy controls	12 months after initiation of treatment t vs. Pre-treatmen
Total score	83.6 ± 12.1	81.9 ± 11.6	*P* 0.301 ES −0.14 [− 0.39, 0.12]	88.5 ± 10.1	*P* 0.024 ES 0.41 [0.15, 0.67]	*P* 0.001 ES 0.61[0.24, 0.97]	83.0 ± 11.1	*P* 0.527 ES − 0.05 [− 0.47, 0.37]	83.5 ± 8.6	*P* 0.469 ES − 0.01 [− 0.43, 0.41]	*P* 0.916 ES 0.06 [− 0.55, 0.67]
Physical Health Domain	85.2 ± 14.8	88.4 ± 10.8	*P* 0.257 ES 0.22 [− 0.04,0.48]	93.8 ± 8.4	*P* < 0.001 ES 0.59 [0.33, 0.85]	*P* 0.002 ES 0.56 [0.2, 0.92]	90.2 ± 8.8	*P* 0.229 ES 0.34 [− 0.08, 0.76]	90.3 ± 9.2	*P* 0.205 ES 0.35 [− 0.08, 0.77]	*P* 0.971 ES 0.01 [−0.6, 0.62]
Psychosocial Health Domain	83.4 ± 13.2	79.7 ± 13.2	*P* 0.029 ES − 0.28 [− 0.54, − 0.22]	86.7 ± 11.7	*P* 0.028 ES 0.25 [− 0.01, 0.51]	*P* 0.002 ES 0.56 [0.2, 0.92]	80.6 ± 12.5	*P* 0.237 ES − 0.21 [− 0.64, 0.21]	81.3 ± 9.9	*P* 0.195 ES − 0.16 [− 0.58, 0.26]	*P* 0.916 ES 0.06 [− 0.55, 0.67]
Emotional Functioning	76.5 ± 18.9	73.2 ± 17.2	*P* 0.156 ES − 0.18 [− 0.43, 0.08]	81.2 ± 17.9	*P* 0.024 ES 0.25 [− 0.01, 0.51]	*P* 0.007 ES 0.46 [0.1, 0.82]	74.3 ± 18.1	*P* 0.535 ES − 0.12 [− 0.54, 0.31]	74.7 ± 15.3	*P* 0.463 ES − 0.1 [− 0.52, 0.33]	*P* 0.953 ES 0.02 [− 0.58, 0.63]
Social Functioning	88.9 ± 15.7	82.7 ± 16.2	*P* 0.003 ES − 0.39 [− 0.65, − 0.14]	88.8 ± 13.5	*P* 0.899 ES − 0.01 [− 0.26, 0.25]	*P* 0.033 ES 0.41 [0.05, 0.77]	83.4 ± 13.4	*P* 0.027 ES − 0.35 [− 0.77, 0.07]	85.2 ± 14.3	*P* 0.182 ES − 0.24 [− 0.66, 0.19]	*P* 0.607 ES 0.06 [− 0.55, 0.67]
School Functioning	84.9 ± 13.6	83.3 ± 14.3	*P* 0.375 ES − 0.12 [− 0.37, 0.14]	90.1 ± 11.2	*P* 0.001 ES 0.39 [0.13, 0.64]	*P* 0.002 ES 0.53 [0.17, 0.89]	84.1 ± 15.3	*P* 0.809 ES − 0.06 [− 0.48, 0.36]	84.1 ± 10.2	*P* 0.722 − 0.06 [− 0.48, 0.36]	*P* 0.596 ES 0 [− 0.61, 0.61]

Scores are presented with SDs, and ESs with their 95% confidence intervals [in intervals]. ES calculations and paired t tests were conducted for for completers at after treatment, by comparing scores with those at baseline

Appendices: ES, effect size; *P*, *p* value; CR, complete response

**Table 4:** Pre- and Post-treatment mean PedsQL scores of patients in groups A and B and of healthy controls: parent-proxy report

	Healthy controls (N = 999)	Group A (*N* = 66)					Group B (*N* = 22)				
		Pre-treatment	Pre-treatment vs. Healthy controls	After CR	After CR vs. Healthy controls	After CR vs. Pre-treatment	Pre-treatment	Pre-treatment vs. Healthy controls	12 months after initiation of treatment	12 months after initiation of treatment vs. Healthy controls	After 12 months of initiation of treatment vs. Pre-treatment
Total score	85.5 ± 13.4	84.4 ± 12.6	*P* 0.376 ES − 0.88 [− 0.33, 0.17]	89.5 ± 10.4	*P* 0.038 ES 0.3 [0.05, 0.55]	*P* 0.012 ES 0.44 [0.09, 0.8]	88.0 ± 9.5	*P* 0.681 ES 0.19 [− 0.24, 0.61]	90.5 ± 11.0	*P* 0.086 ES 0.37 [− 0.05, 0.8]	*P* 0.915 ES 0.24 [− 0.37, 0.85]
Physical Health Domain	86.2 ± 19.9	87.7 ± 14.6	*P* 0.812 ES 0.08 [− 0.18, 0.33]	94.2 ± 9.0	*P* 0.013 ES 0.41 [0.15, 0.67]	P 0.012 ES 0.54 [0.19,0.89]	94.5 ± 12.9	*P* 0.062 ES 0.42 [0, 0.84]	95.8 ± 7.6	*P* 0.024 ES 0.49 [− 0.06, 0.91]	*P* 0.907 ES 0.12 [− 0.49, 0.73]
Psychosocial Health Domain	85.2 ± 13.2	83.4 ± 13.8	*P* 0.314 ES − 0.14 [− 0.39, 0.12]	87.9 ± 11.2	*P* 0.127 ES 0.21 [− 0.05, 0.46]	*P* 0.061 ES 0.37 [0, 0.72]	86.2 ± 10.6	*P* 0.972 ES 0.08 [− 0.35, 0.5]	88.7 ± 12.9	*P* 0.202 ES 0.27 [− 0.16, 0.69]	*P* 0.211 ES 0.21 [− 0.4, 0 82]
Emotional Functioning	84.5 ± 14.9	82.8 ± 15.2	*P* 0.365 ES − 0.11 [− 0.37, 0.14]	88.3 ± 11.2	*P* 0.135 ES 0.26 [0, 0.52]	*P* 0.057 ES 0.41 [0.05, 0.77]	81.3 ± 14.9	*P* 0.293 ES − 0.21 [− 0.64, 0.21]	89.5 ± 10.9	*P* 0.235 ES 0.34 [− 0.09, 076]	*P* 0.077 ES 0.63 [0, 1.25]
Social Functioning	86.5 ± 17.4	82.6 ± 21.2	*P* 0.237 ES − 0.22 [− 0.48, 0.04]	87.4 ± 16.0	*P* 0.687 ES 0.05 [− 0.2, 0.31]	*P* 0.269 ES 0.26 [− 0.1, 0.61]	88.9 ± 17.5	*P* 0.47 ES 0.14 [− 0.28, 0.56]	87.9 ± 18.5	*P* 0.825 ES 0.08 [− 0.34, 0.5]	*P* 0.929 ES − 0.06 [− 0.66, 0.55]
School Functioning	84.6 ± 14.2	84.6 ± 13.7	*P* 0.932 ES 0 [− 0.26, 0.26]	88.2 ± 12.7	*P* 0.055 ES 0.25 [0, 0.51]	*P* 0.134 ES 0.27 [− 0.08, 0.63]	88.4 ± 12.7	*P* 0.212 ES 0.27[− 0.15, 0.69]	88.7 ± 13.9	*P* 0.127 ES − 0.29 [− 0.13, 0.71]	*P* 0.594 ES − 0.02 [− 0.59, 0. 63]

Scores are presented with SDs, and ESs with their 95% confidence intervals [in intervals]. ES calculations and paired t tests were conducted for for completers at after treatment, by comparing scores with those at baseline

Appendices: ES, effect size; *P*, *p* value; CR, complete response

Based on the parent proxy reports, the mean PedsQL total scale score (84.4 ± 12.6 vs. 89.5 ± 10.4; *p* = 0.041, effect size 0.44) and physical health summary score (87.7 ± 14.6 vs. 94.2 ± 9.0; *p* = 0.012, effect size 0.54) were significantly improved after achievement of CR compared to the pre-treatment scores in group A. On the other hand, no significant improvement in the mean PedsQL total scale score, physical health summary score, psychosocial health summary score, or other functioning domain scores were observed in group B based on the parent proxy-reports (Table 4).

## Discussion

We evaluated the HRQOL of children with DUI using the PedsQL questionnaire to assess the physical and psychosocial health and the adaptability of the children in their daily lives. Previous studies that assessed the HRQOL of children with lower urinary tract dysfunction (such as DUI and nocturnal enuresis) employed various instruments, including the Pediatric Incontinence Questionnaire [28], the DISABKIDS chronic generic measurement [29], and the PedsQL questionnaire [30, 31]. Questionnaires for assessment of HRQOL should ideally be multidimensional, subjective, and quantitative [10]. However, when the respondents are young children, it may be difficult to collect valid self-ratings and complete questionnaire responses. Therefore, inclusion of parent’s proxy-reports of HRQOL provides valuable complementary information in addition to that provided by the affected child’s self-reports [32]. Children may sometimes report their HRQOL to be very different from that reported by their proxy; in such a situation, there is no objective method to determine whether the views of the child or the proxy reflect the “truer” picture. The questionnaires used to evaluate the quality of life of patients with lower urinary tract symptoms in previous studies (except PedsQL) were only based on child self-reports, and did not include the parent’s proxy-reports. We believe that the main advantage of the PedsQL is that it allows for assessment of the HRQOL of children with DUI based on both child self-reports and parent proxy-reports. In addition, the questionnaire has good psychometric properties (including social and school functioning) among healthy populations as well as children with chronic conditions.

The presence of DUI negatively impacts the daily life of children, which may affect their relationships with their peers and cause learning difficulties, parental anxiety, and punitive actions since incontinence episodes can occur at school and during social activities [8, 17, 33]. The impaired HRQOL of children with DUI observed in our study is consistent with that reported in previous results [13, 21]. In a study conducted in Sweden by Gladh et al. [13], children with DUI were found to have a poorer quality of life than healthy children. Problems associated with DUI were also found to influence the social life, self-esteem, and self-confidence of the affected children. Our results also showed that DUI has a negative impact on the HRQOL of children; in addition, achievement of CR was associated with improved HRQOL. Schast et al. [30] used the PedsQL to assess the HRQOL of 351 American children who were referred to a special voiding clinic; the mean total scale score of children based on self-reports was 82.9, which is similar to our finding in Japanese children with DUI. However, Veloso et al. [31] reported that the mean total PedsQL scale score was 71.0 in Brazilian children and that the mean score for the school functioning domain (54.8) was lower than that observed in the present study. Various social and environmental affect the HRQOL of children, including parental income, residential setting (urban or rural), and level of parental education [10]. One possible explanation for the difference in mean scores between the study conducted by Veloso et al. [31] and our study is the differences with respect to social background, race, income, and the developmental environment. Further research is needed to identify whether differences in social background, race, or income affect not only the total HRQOL scores, but also subdomain scores in the PedsQL.

After achievement of CR, group A patients had higher scores than those of the healthy controls; this may reflect that the self-image and self-esteem of patients were seriously affected by DUI, and that the resolution of DUI relieved the psychological burden and greatly increased the quality of life.

We observed a significant improvement in child self-reported and parent proxy-reported HRQOL in children with DUI after treatment. The observed improvement in the HRQOL of these children is consistent with that observed in previous studies. In one such study, a group of children with DUI who responded to treatment showed significant positive changes in HRQOL after 3-month treatment, as assessed by the German version of the Pediatric Incontinence Questionnaire [21]. The results of the present study showed significant post-treatment improvement in scores for all items of the PedsQL based on the child self-reports. Post-treatment scores other than those for social functioning and school functioning also showed a significant improvement based on the parent proxy-reports.

However, it is unclear if this improvement in the HRQOL was in fact the result of undergoing treatment for DUI or the result of resolution of DUI. We therefore considered it necessary to identify the factors that contributed to the improvement in HRQOL. Therefore, we categorized subjects into two groups (those that achieved CR within 12 months and those that did not achieve CR within 12 months) to verify the degree of improvement in HRQOL. In our previous study of the duration of therapy for DUI with overactive bladder, the mean duration to achieve CR for DUI was 11.9 months [34]. Therefore, patients were divided into two groups: those who achieved CR within 12 months and those who achieved after > 13 months.

Our rationale for the use of CR as an index for grouping was that the impact of the study conducted by Equit et al. [21] on the HRQOL of children could not be ruled out if DUI episodes persisted several times a week despite children showing a positive response to treatment. We also considered that evaluation of the difference between the HRQOL scores of patients and those of healthy controls in addition to the degree of improvement in HRQOL at the time of CR would enable more accurate evaluation of changes in the HRQOL due to complete resolution of DUI compared with the period when DUI was present. We found that patients who achieved CR within 12 months (group A) showed significant improvement in all items after achieving CR compared to the respective pre-treatment levels. Furthermore, social functioning, which was significantly lower than that of healthy controls prior to initiation of treatment, showed no significant difference with that of healthy controls after achievement of CR. We showed that the reduced social quality of life of children, such as impaired friendship, becomes comparable to that of healthy children after resolution of DUI. Even more surprising is the fact that the physical health summary score, psychosocial health summary score, and emotional and school functioning domains were all significantly higher than those of healthy controls after achievement of CR. The post-treatment total scale score was also significantly higher than that of healthy controls. This demonstrated that children who experienced disruption of their school life due to DUI (e.g., soiling of clothes and the consequent restrictions to their activities due to the associated discomfort and embarrassment) exhibited increased activity after resolution of DUI and showed greater improvement than healthy controls with respect to their physical functioning, emotional functioning, and school life.

However, patients that did not achieve CR (group B) exhibited no post-treatment improvement in the HRQOL associated with social functioning, which had declined significantly before initiation of treatment. Within the scope of our investigation, we did not encounter any previous studies that examined the impact of CR on the HRQOL of children with DUI.

Based on the parent proxy-reports, we observed significant post-treatment increase in physical health scores both in group A and group B; however, there were no significant between-group differences with respect to the total scale score or the improvement in the psychosocial quality of life. Furthermore, based on child self-reports, both groups showed a significant improvement in social quality of life after achievement of CR; however, this phenomenon was not observed in either groups based on the parent proxy-reports. This suggests that parents may not notice the adverse effects of the presence or absence of DUI on their child’s social quality of life.

Subsequently, we focused on the differences domain scores between the child self-reports and the parent proxy-reports of the PedsQL. In the comparison between the healthy controls and cases, the social functioning domain scores were significantly lower for the cases than for the healthy control in the child self-report; however, the same finding was not observed for parent proxy-reports. These marked differences between the parent proxy-reports and child self-reports may be due to parents’ overlook, leading to deterioration of the social functioning of children with DUI. For example, these children occasionally experience difficulty in building friendships. Physicians and healthcare providers should support such children to ensure that they can perform well in their schools and make friends because their parents tend to be unaware of these problems.

With regards to the pre- and post-treatment PedsQL scores, the emotional functioning domain scores were significantly elevated in group A in child self-reports; however, no significant increase was observed for parent proxy-reports. This suggests that parents are unaware and are missing out on the improved emotional functions in their children by eliminating DUI.

Moreover, even if DUI is eliminated, parents may still showcase negative feelings toward their children. Physicians and health care providers need to be aware that this behavior of parents toward their children can also lead to a lack of parental support and impact the outcomes of children receiving therapy for DUI, as supported by the recommendation of ICCS.

We believe that the aforementioned results are noteworthy because they demonstrate that DUI itself has a major impact on the physical and psychosocial functioning of children and that the resolution of DUI after treatment may help improve the physical and school functioning of these children and lead to a higher quality of activity and school-related quality of life than healthy children. Furthermore, we believe it is necessary to aim for an early cure (within 1 year of onset) in order to help improve the HRQOL of children with DUI.

Five main limitations of this study should be considered while interpreting the results. First, the quasi-experimental nature of the study precludes the element of random assignment. Second, data pertaining to patients were collected both before and after treatment, but only once in the control group. Third, only 71% of recruited patients were included in the analysis; the effect of exclusion of 29% patients on our results cannot be ruled out. Forth, it was not possible to understand the clinically significant point of difference and SD because there were no available previous reports on the PedsQL score in children with lower urinary tract symptoms such as DUI. Therefore, we did not calculate the sample size before starting the study. Finally, This study did not examine the individual factors of children affecting these outcomes. Therefore, we plan to interview these children to understand their friendship and school life because it is necessary to clarify the individual psychological stress factors of children with DUI. In particular, we aim to clarify individual factors by conducting semistructured interviews on episodes of difficulties in school life and poor friendships.

## Conclusions

Although several studies have reported that improvement in HRQOL can be achieved after treatment of DUI [22, 23, 35], the results of our study suggest that the impact of the resolution of DUI on improving HRQOL is greater that the impact of a decrease in the frequency of DUI. Moreover, the resolution of DUI relieved the psychological burden and greatly increased the quality of life. Therefore, it is important to actively treat with children with daytime incontinence to achieve complete resolution of DUI.

## Data Availability

All data generated or analyzed during this study are included in this published article.

## Abbreviations

CR: Complete response
DUI: Daytime urinary incontinence
DVSS: Dysfunctional voiding symptom score
HRQOL: Health-related quality of life
ICCS: International Children’s Continence Society
PedsQL: Pediatric Quality of Life Inventory 4.0
